# Effect of non-invasive brain stimulation in children with acquired brain injury—a scoping review

**DOI:** 10.3389/fneur.2024.1388718

**Published:** 2024-08-29

**Authors:** Chandrasekar Rathinam, Vikram Mohan, Derick Yates, Peter Bill, Janet Peirson, Rajat Gupta

**Affiliations:** ^1^Birmingham Medical School, University of Birmingham, Birmingham, United Kingdom; ^2^Birmingham Women’s and Children’s NHS Foundation Trust, Birmingham, United Kingdom; ^3^Department of Rehabilitation and Sport Sciences, Faculty of Health and Social Sciences, Bournemouth University, Bournemouth, United Kingdom; ^4^Retired Physiotherapist, Cambridge, United Kingdom; ^5^School of Pharmacy, Aston University, Birmingham, West Midlands, United Kingdom

**Keywords:** acquired brain injury, children and young people, transcranial magnetic stimulation, transcranial direct current stimulation, motor function, TMS, tDCS

## Abstract

**Background:**

Children and young people (CYP) with acquired brain injury (ABI) require early and effective neurorehabilitation to improve long-term functional outcomes. Non-invasive brain stimulation (NIBS), including transcranial magnetic stimulation (TMS) and transcranial direct current stimulation (tDCS), have been used to improve motor and sensory skills for children with cerebral palsy. However, there is limited evidence supporting its use in CYP with ABI.

**Objective:**

To systematically review the TMS and tDCS intervention effects on motor, sensory and other functional issues in CYP with ABI as reported in the literature.

**Methods:**

A comprehensive online bibliographic databases search was performed in various databases using keywords related to NIBS and CYP with ABI. Studies that examine the effect of NIBS intervention on motor function and other functional difficulties either as a primary or secondary objective were included in this review.

**Results:**

Fourteen studies (10 single case reports, one retrospective analysis, one case series, one randomised and one quasi-randomised controlled trial) published between 2006 and 2023 were identified. These studies examined the use of NIBS to manage motor disorders, hearing, vision, headaches, speech and language and memory issues. Seventy-six children with mild to severe ABI had received NIBS. The session frequency (3–20), duration (10–45 min) was variable, and NIBS delivered between 3 and 28 days.

**Conclusion:**

The literature describing NIBS interventions in CYP with ABI is scarce. An insufficient number of studies, inadequate information reported in them, and small sample sizes limit the ability to conclude how effective NIBS is in improving motor function and other functional issues in this cohort. Further studies are therefore necessary to examine the therapeutic effects of NIBS to manage various functional problems in the CYP with ABI.

## 1 Introduction

Acquired brain injury (ABI) is the term used to describe traumatic and non-traumatic brain injuries that occur after birth and a period of typical development (1). Children and young people (CYP) with severe ABI will often have movement difficulties caused by weakness, abnormal muscle tone, poor motor control, poor concentration, poor sensory regulation, fatigue and other comorbidities (2). They may also have difficulties with speech, swallowing, and cognitive impairment. During the acute and sub-acute phase, CYP with moderate to severe ABI frequently require a period of demanding medical and rehabilitative care to optimise their long-term capabilities and quality of life through neuroplasticity (3).

Early and effective neurorehabilitation provision promotes a good long-term functional outcome for CYP with ABI (4). Recent advances in technology enable clinicians to use functional electrical stimulation, virtual reality (5), and non-invasive brain stimulation (NIBS), which includes transcranial magnetic stimulation (TMS), transcranial direct current stimulation (tDCS), transcranial alternating current stimulation (tACS), transcranial ultrasound stimulation, vagus nerve stimulation, and galvanic vestibular stimulation (6) to help improve motor skills, tone management, and sensory problems of children with central nervous system disorders (7, 8).

TMS is a non-invasive, safe treatment technique. It delivers repetitive magnetic pulses directly to specifically targeted brain areas through electromagnetic induction. Low-frequency TMS reduces cortical excitability, but high-frequency increases it (9). TMS stimulates neurons in the brain through depolarisation of myelinated axons, excites inhibitory interneurons in the stimulated brain area, and propagates along axons and synapses (10). Navigated repetitive TMS is delivered to a targeted brain area to change polarisation, influencing cortical excitability many minutes after initial stimulation (11). This will help to facilitate, inhibit or interrupt the cortical network depending upon the frequency and intensity of the stimulus, thus promoting a cortical function change through neuroplasticity (12). TMS has been used to treat children with cerebral palsy (CP) (13) and neuropsychiatric disorders, including children on the autistic spectrum and those with attention deficit hyperactivity disorder, obsessive-compulsive disorder, and tics (7). It has been shown to improve gait and upper limb functions due to changes in body structure and function, and activities in CYP with CP (14).

tDCS is another type of NIBS in which a low-amplitude current stimulates the brain by modulating neural activities (15). In tDCS, the electrodes are placed on the scalp, and the current enters the brain by passing between electrodes. The electrical activities within the brain either inhibit or facilitate neural activities to produce therapeutic effects (16). Neuronal depolarisation occurs in anodal stimulation and hyper-polarisation in cathodal stimulation by changing the membrane potential. tDCS has been used in adults and CYP with various neurological disorders including multiple sclerosis, stroke, and CP, improving motor function and fatigue, thereby improving their participation (14, 17). tDCS influences the regulatory mechanism on motor and cognitive functions through changes in the neurotransmission system, synaptic microenvironment and neural connectivity (18).

Neural plasticity (or neuroplasticity), a mechanism where the brain dynamically responds to the environment and the experience through changes in neural circuits, is critical to influencing cognitive and behavioural development (19). In ABI, the neural network is destructed or disrupted due to neuronal death, axonal tract damage, glia function, changes in the neurotransmitter system and poor cerebral perfusion. The destructed neurons and cell bodies are not replaced, and the injured axons grow slowly or minimally through regeneration. Glial responses to injury involve repairing and preserving the existing cell population through glial scar formation, and regenerating lost populations, including neurons and glia (20, 21). The molecular changes within the injured brain cells disrupt gene expression and protein phosphorylation, which are responsible for effective nerve conduction and regulation of neuron protein components. These changes are detrimental to the injured brain as they alter or interrupt normal brain development. Consequentially, children with ABI tend to have slower development or emerging long term functional problems (22). The brain’s response to environmental stimulation, a key component of neural plasticity, is limited in CYP with ABI due to the brain injury itself and the consequential impact this has on the CYP’s abilities to respond to environmental stimulation through participation. This then further restricts their recovery and development.

Interventions that promote neural plasticity in the developing brain may be advantageous in rehabilitation following ABI. It is considered that NIBS facilitates structural and functional neural plasticity through changes in regional volumes in brain cells or the formation of neural pathways through synaptogenesis, axonal or dendritic sprouting, and the creation of new neurons (23–25). If this is the case, NIBS combined with intensive rehabilitation appears to be a promising new interventional approach with broader future applications for CYP with ABI. There is, however, limited material supporting its use in CYP with ABI.

The intervention effect in rehabilitation research has been widely reported using the International Classification of Functioning, Disability and Health for Children and Youth (ICF-CY) framework (26). The ICF-CY domain consists of body structures and function, activity, participation, and contextual factors (environment and personal), which can be used to classify the level of functioning in childhood (13). This model can be applied to report the functional outcome of CYP with ABI who have impaired physical, cognitive and emotional difficulties and their impact on activity limitation and participation restriction following an intervention (13). To our knowledge, no review has examined the therapeutic effect of TMS and tDCS in CYP with ABI using the ICF-CY framework. This is a gap that should be addressed.

## 2 Methods

A brief method description is given here. The detailed methodology can be found in the protocol (27) and the deviations in the open-access framework.^1^ This review followed the Preferred Reporting Items for Systematic Reviews and Meta-Analysis extension for Scoping Reviews (PRISMA-ScR) checklist (28) (Supplementary File 1) and Arksey and O’Malley’s (29) Five stage scoping review approach.

### 2.1 Stage 1: identifying the research question

The purpose of this scoping review was to examine the literature relating to NIBS therapeutic effects in any functional domains such as motor, sensory and cognition. We have included only TMS and tDCS for this review to denote NIBS. The outcomes of this review were categorised according to the ICF-CY dimensions.

### 2.2 Stage 2: identifying relevant studies

Comprehensive online bibliographic searches were performed in Medline, Embase, Emcare, Cinahl, AMED and Cochrane Central databases by the professional librarian (DY). Results were screened for duplicate citations and remaining abstracts were uploaded in the Ryaan software (30). A copy of the full search strategy as run in Ovid Medline is provided in Appendix 1. Hand searches of citations, article references, conference proceedings identified by the searches, and trial registers checking were also carried out. The review team also contacted various TMS equipment manufacturers and distributors.

### 2.3 Stage 3: study selection

**Figure 1:**
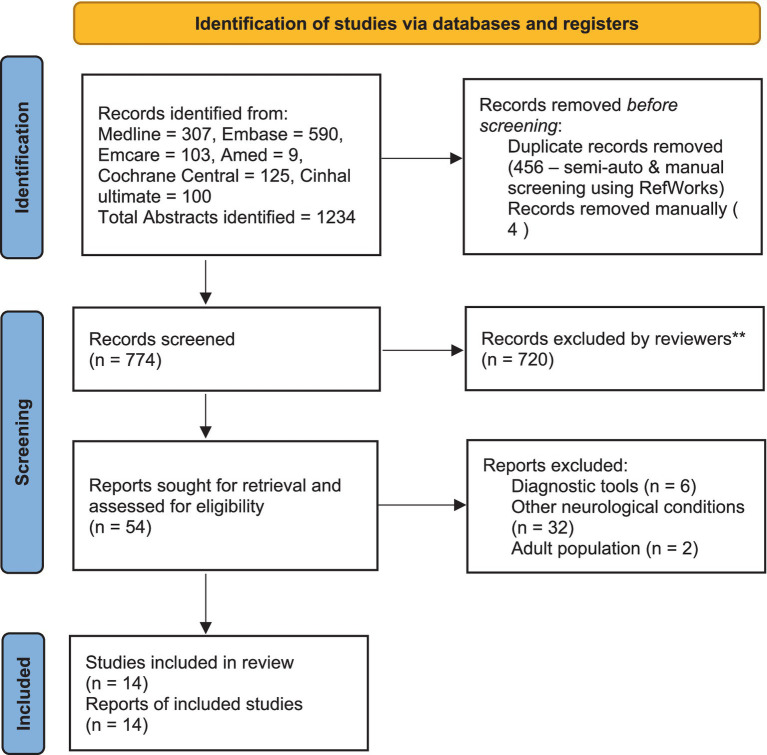
PRISMA flow chart diagram describing the study selection process.

Two reviewers (CR and VM) scrutinised the collected titles and structured abstracts based on the following criteria. The searches were confined to CYP with ABI only (age group 2–18 years). All the subgroups of ABI, including traumatic, non-traumatic and brain tumour, were included, but children with CP were excluded. Research studies that include TMS for diagnostic purposes were excluded. No exclusion criteria were set for language or publication years, and these studies were considered if the title and abstracts had been written in English. Full articles that met the selection criteria from the above source were collected. If the full report and conference abstract were available for the same study, only the full report was included. Two reviewers decided which articles were suitable for the final review, and any disagreements were managed after discussion with the third reviewer (RG). A PRISMA flowchart was used to inform the selection process (Figure 1).

Our search yielded 1,234 titles and after removing duplicates, 774 studies were scrutinised. Fifty-four full-text reports (articles and abstracts) were collected, and 14 studies were included in the final review. Of these, 10 were single case reports, two were randomised controlled trials (RCT), one was a case series (*n* = 2), and one was a retrospective cohort analysis (*n* = 44). The total number of participants from all these studies was 76 (2–18 years). Five studies used TMS (*n* = 25), and nine used tDCS (*n* = 51).

### 2.4 Stage 4: charting the data

After the screening, two reviewers (CR and VM) independently extracted the data, and the details of which are given in Table 1. All the outcomes reported in the selected articles were classified under ICF-CY domains (Table 2).

**Table 1:** Summary of the studies reporting TMS and TDS effects in children with acquired brain injury

Authors Year and Country	Study design	Patient demographic information	Diagnosis and ABI type	Non-invasive brain stimulation procedure			Associated therapy	Outcome measures	Reported therapeutic effect
				Technique, stimulation area	Stimulation parameters	adverse effects			
Kirton A et al. 2008 Canada (31)	Parallel, randomized trial (full paper)	Median age 13.25 [Inter Quartile Range 10.08–16.78] years Mean time post-stroke 6.33 [Std. Dev 3.56] years) n = 10 (2 were 20 years old)	Stroke Non-traumatic ABI	Repetitive TMS Sham treatment or inhibitory, low-frequency over contralesional motor cortex (1200 stimuli)	Once per day for 8 days 20 min/session	No serious adverse events Single episode of neurocardiogenic syncope with initial exposure to repetitive TMS (n = 2) Mild headache (n = 2) Nausea and neck stiffness (n = 1)	None reported	Grip strength Melbourne assessment of upper extremity function (MAUULF)	Improvement in grip strength at day 10 but did not persist to day 17
Niimi M et al. 2013 Japan (32)	Case Series Study (full paper)	8 and 9 years old Male Age at injury: 2 years n = 2	Stroke and upper limb hemiparesis Non-traumatic ABI	High Frequency repetitive TMS High Frequency Repetitive TMS (10 Hz) (total 1500 pulses) to the primary motor areas of lesional hemisphere Stimulation intensity - 90% of the motor threshold of the first dorsal interosseous muscle	22 sessions over 15 days 15 min/session	No adverse effects	30-min one-to-one Occupational therapy and 30-min self-exercise	Motor function assessment using Fugl-Meyer Assessment, Wolf Motor Function Test, and Simple Test for Evaluating hand Function	Child-1: Marked improvement in endurance during grasping a bowl; Able to throw a small ball more powerfully Child-2: Handle chopsticks, write better by holding the pen, use hand more frequently in activities of daily living, move fingers more smoothly to play piano with the affected hand Improvement persisted up to 4 weeks
Marei A et al. 2017 Egypt (33)	Single case report (Conference abstract)	17-year-old Female Age at injury: 17-year-old	N-Methyln-D-Aspartate Receptor encephalitis Non-traumatic ABI	Repetitive TMS Two protocol: (a) 30 pps,100 pulse/train, 20 trains, 2 sec inter train interval, intensity10%, over the scalp (b) 5 pps,100 pulse/train, 20 trains, 20 sec inter train interval, intensity 50% over the dorsolateral prefrontal cortex	5 consecutive sessions for 4 weeks Session length not reported	Not reported	Occupational Therapy sessions (1^st^ week - 5 sessions; subsequent weeks 3 sessions)	Wechsler Memory scale (cognitive functions)	Improvement of the motor symptoms Improvement in cognitive functions improvement in Mental Control domain
Carlson H et al. 2016 Canada (39)	Single case report (full paper)	17-year-old Male Age at injury: 15 years	Stroke Moderate-severe non-fluent dysphasia; Right homonymous hemianopsia and severe hemiparesis Non-traumatic ABI	Repetitive TMS Right inferior frontal gyrus (IFG) pars triangularis at 1 Hz with an intensity of 120% Resting Motor Threshold (1200 stimuli)	10 days over 2 weeks 20 min/session	Uncomfortable facial twitching on the first day No adverse events such as headaches, neck pain or unpleasant tingling reported	Customized Speech and Language Therapy program	Language assessments (Language function - Boston Diagnostic Aphasia Examination-Short-3; Daily picture naming accuracy) Imaging [Task functional Magnetic Resonance Image (MRI), Resting state functional MRI, Diffusion imaging, Magnetic resonance spectroscopy]	Clinically significant changes in expressive language function (overall fluency, phrase length, and articulation of speech sounds); confident in speaking and improved activities of daily living
Anninos P et al. 2006 Greece (34)	Single case report (full paper)	2-year- old Male	Epstein- Barr virus (EBV) encephalitis Mutism, ataxia and loss of the ability to eat, walk and talk Non-traumatic ABI	TMS sessions Area of stimulation, specification not reported	2 times a week for 3 years Session length not reported	Not reported	Not reported	Magnetoencephalography map of the right temporal area biomagnetic values at the beginning < 1600 Ft/Hz; after 2 years < 1800 Ft/Hz, after 3 years > 2200 Ft/Hz	Improvement of the clinical findings reported but no specific details given
Saleem G et al. 2021 USA (44)	Single case report (Conference abstract)	15-year-old Female	ABI and Spastic quadriplegia Type of ABI - Unknown	Repetitive tDCS (sham, 1 mA, and 2 mA) over the dorsolateral prefrontal cortex	Three sessions 20 min/session	Severe itching at the anodal site with 2 mA stimulation and erythema	No specific information available	Digit Span Test	No specific information available
Pinchuk D et al. 2013 Russia (42)	Retrospective cohort study (Full paper)	Age range (median): 11–16 years Male and female participants n = 44	Migraine headache Traumatic ABI (mild)	tDCS 60 - 90mA intensity First electrode position (1EP): anode - frontal pole of the hemisphere sub- dominant in motor skills; cathode - ipsilateral mastoid process [n = 38] Second electrode position (2EP): anode - center of the forehead; cathode - 2 cm higher the mastoid process of the hemisphere subdominant in motor skills [n = 6]	5 – 9 sessions with 4 – 7 days interval 30 – 45 min/session	No negative side effects	Not reported	Headache frequency (days/month), Headache severity scale (0–10), % patients with ≥50% reduction in headache frequency, % patients with headache resolution	First electrode position - Headache resolution (52%) and reduction of headache at least half (29%); total improvement 81%; no effect 19% Second electrode position - Reduction of headache at least half (33%); no effect 66% Significant improvement in headache severity and frequency
Ryan J et al. 2023 Canada (35)	Single case report (full paper)	Adolescent (age not given) Male Age at injury: Not given	Right hemiparesis and hypertonia Traumatic ABI	tDCS Intensities - between 1.0 and 2.0 milliamperes (mA) Anode over Cz (i.e., the lower extremity region of bilateral primary motor cortices) Cathode over Fpz (i.e., center of the forehead)	16 sessions over 23 days 20 min/session	Itchy (one session) and tired (two sessions)	Physiotherapy Occupational therapy Speech language pathology	Gross Motor Function Measure (GMFM-88) COPM 10-metre Fast Walk Test Parent-reported Pediatric Evaluation of Disability Inventory Goal Attainment Scaling	Mobility progressed from wheelchair use to ambulation with a walker post-intervention GMFM score increased 33.1% Session tasks often had several foci (e.g., skill acquisition, strength, and balance) with task focus changing as the youth progress
Mori T et al. 2016 Japan (40)	Single case report (full paper)	13-year-old Female Age at injury: 6 years	Brainstem encephalitis bilateral hearing impairment Non-traumatic ABI	tDCS (1 mA) Anode - left auditory cortex Cathode - contralateral supraorbital region	Once a day for 4 consecutive days 10 min/session	No adverse side-effects	None	Pure tone audiometry and speech audiometry tests Speech discrimination tests	Speech discrimination in the right ear improved but not in left ear; treatment effect lasted 4 days after intervention No changes in pure tone audiometry in ears
Ciechanski P et al. 2020 Canada (36)	Single case report (full paper)	17-year-old Female Age at injury: 6 years	Arterial ischemic stroke hemiparesis in the upper extremity Non-traumatic ABI	tDCS Cathode – contralesional M1 hotspot Anode - ipsilesional supraorbital region Stimulation current was ramped to 1.5 mA over 45 seconds, and ramped-down over 45 seconds	10 consecutive week days 20 min/session	Mild tingling and itching during the ramp-up phase, typically subsiding within 3 minutes. No additional side effects reported	Daily intensive rehabilitation therapy (Occupational Therapy, gross and fine motor skill training)	Jebsen Taylor Hand Function Test Assisting Hand Assessment Box & block test Grip strength Melbourne assessment of upper extremity function COPM	Jebsen Taylor Hand Function Test - 44% improvement from baseline Assisting Hand Assessment - Significant increase (≥5 points) COPM (≥2 points), mean goal performance (2/10 at baseline to 8/10 post-intervention) and accuracy and fluency measures Motor outcomes sustained at 6 months
Nagai M et al. 2018 Japan (37)	Single case report (full paper)	8-year-old Male Age at injury: 2 years	Hypoxic encephalopathy Non-traumatic ABI	tDCS Cathodal or sham tDCS 1mA using a DC stimulator Cathodal - supplementary motor area (SMA), Anodal - left supraorbital region	3 sessions, at least 2 days apart 10 min/session	No adverse side-effects	No	Accelerometer attached to the forehead	Decreased involuntary movements of the head and neck during standing
Kamali A et al. 2021 Iran (41)	Single case report (full paper)	11-year-old Female Age at injury: 6 years	Ultra-low vision following occipital ischemic insult and ischemic optic neuropathy Non-traumatic ABI	tDCS and transorbital Alternating Current Stimulation (tACS) (1) tDCS - anodes placed on the left and right occipital area and cathodes on the left and right shoulders using a 2 mA (2) tACS - electrodes positioned at FP1 and FP2, and bilaterally on maxilla using a 25 Hz, 1.5 mA	5 sessions of tDCS (25 minutes) followed by tACS (25 minutes) over five consecutive days	Not reported	Sodium valproate, Modafinil and PreserVision 3 Self-training eye exercise named Fit Eye	Best Corrected visual acuity Low Vision Quality of Life questionnaire	Sustained improvement in visual function (over 20 months follow-up)
Batista and Porto 2019 Brazil (38)	Single case report (Conference abstract)	18-year-old Male Age at injury: 13 years	Head-shot accident Traumatic ABI	tDCS Anodic - stimulation of cerebellum Cathode - supraorbital and bi anodic stimulation of motor areas Intensity of 2mA	15 consecutive sessions plus sessions once a week for 6 weeks 20 min/session	Not reported		No specific outcome measures reported	Gait improvement with dissociated upper limb movements Speech improvement with the presence of dysarthria and articulation difficulties Attention and memory improvement with beginning of reports of daily activities and reduction of hypersensitivity Returned to use consoles, computers and increased participation in the professional stage
Quinn de Launay K et al. 2022 Canada (43)	Quasi-randomized pilot trial (full paper)	13–18 years 10 females 2 males (n = 6 in experimental group and n = 6 in control group)	Persistent post-concussion symptoms Traumatic ABI	tDCS Experimental group 1.5 mA Control group (Sham) tDCS Anode - left Dorsolateral prefrontal cortex (DLPFC) area Cathode - right supraorbital region	3 sessions 20 min/session	None to moderate range: Itching, pain, burning, warmth/heat, pinching, metal/iron taste, fatigue, headache, nausea, dizziness Considerable to strong range: Itching, pain, burning, pinching, headache nausea and dizziness	None	Cognitive performance: N-Back experimental task to measure accuracy and reaction time on working memory tDCS adverse effects questionnaire	Enhancing working memory performance Increases in accuracy from Day 1 to 3 seen in both groups

TMS – Transcranial magnetic stimulation; ABI – Acquired brain injury; tDCS – Transcranial direct current stimulation; COPM - Canadian Occupational Performance Measure; mA – milliampere.

**Table 2:** International Classification of Functioning, Disability and Health—Children and Youth domains related outcome reported in the included studies.

Authors	Study type	NIBS type	ICF-CY domains		
			Body structure and function	Activities	Participation
Kirton A et al. (2008) (31)	Parallel RCT	TMS	X		
Niimi M et al. (2013) (32)	Case series	TMS		X	X
Marei A et al. (2017) (33)	Single case report	TMS	X		
Carlson HL et al. (2016) (39)	Single case report	TMS	X		
Pinchuk D et al. (2013) (42)	Retrospective study	tDCS		X	X
Ryan J et al. (2023) (35)	Single case report	tDCS	X		X
Mori T et al. (2016) (40)	Single case report	tDCS	X		
Ciechanski P et al. (2020) (36)	Single case report	tDCS	X	X	X
Nagai M et al. (2018) (37)	Single case report	tDCS	X		
Kamali A et al. (2021) (41)	Single case report	tDCS and tACS	X		
Batista and Porto (2019) (38)	Single case report	tDCS	X	X	X
Quinn de Launay K et al. (2022) (43)	Quasi RCT pilot	tDCS	X		

NIBS, Non-invasive brain stimulation; TMS, transcranial magnetic stimulation; tDCS, transcranial direct current stimulation; tACS, transorbital Alternating Current Stimulation.

## 3 Results

### 3.1 Stage 5: collating, summarising, and reporting the results

The TMS intervention had been utilised only in CYP with non-traumatic ABI, and the participants had undergone 8 to 22 TMS sessions over 8 to 28 days (15–20 min/session). The tDCS intervention had been utilised in both traumatic and non-traumatic ABI populations, and they had 3 to 16 sessions over 3 to 23 days, with the length of each session ranging between 10 and 45 min. Variable treatment parameters were used for TMS and tDCS, the details of which are given in Table 1.

### 3.2 Motor function

Three of the four studies reported improved motor function using TMS (31–33). Kirton et al. (31) stimulated the contralateral motor cortex with low-frequency TMS, but Niimi et al. (32) stimulated the ipsilateral primary motor cortex with high-frequency TMS. Both studies had shown improved upper limb motor functions. The reviewers could not extract specific details from the remaining two studies (33, 34). Four single case studies reported improved motor functions following tDCS intervention (35–38). Ryan et al. (35) and Ciechanski et al. (36) study participants had received intensive physiotherapy and occupational therapy along with TMS and tDCS, respectively. Therefore, it is not possible to determine the actual effectiveness of TMS and tDCS in improving motor function.

### 3.3 Speech and language function

Two case studies reported improved speech and language function using NIBS. TMS was used in one of the studies, and the authors attributed combined TMS with intensive speech and language therapy for the observed improvement (39). Batista and Porto (38) reported improved speech articulation using tDCS, but no specific information is available to describe the patient’s baseline speech level and how the authors measured the improvement.

### 3.4 Sensory (hearing and vision)

Mori et al. (40) noted improved hearing in the right ear using tDCS targeting the auditory cortex but found no change in hearing in the left ear. Kamali et al. (41) used combined tDCS and transorbital alternating current stimulation along with visual rehabilitation therapy to treat cerebral visual impairment. The authors noted improved visual acuity and suggested that tDCS is an alternative or adjunct therapeutic approach to managing visual impairment.

### 3.5 Headache

Pinchuk et al. (42) retrospective study examined the effectiveness of tDCS in post-lesional migraine headaches in adults and children. In this review, only data related to CYP was retrieved. The authors used two electrode positions (1EP and 2EP—description in Table 1) to treat headaches. They found that 52% of children in the 1EP group had entirely resolved headaches, and 28% of children’s headaches had reduced by at least 50% after 4.5 months. The authors noted no effect of tDCS for the remaining 20% of participants. This study observed a marked reduction in headache, tiredness, and irritability, and the children had better sleep following tDCS treatment.

### 3.6 Cognitive function

In a single case report, Carlson et al. (39) treated cognitive function with TMS, but no specific information is available to inform the baseline and the level of improvement made. Similarly, Batista and Porto (38) reported improved attention and memory following tDCS intervention, but no specific details are available. Quinn de Launay et al. (43) examined the effect of tDCS on the adolescent population’s cognitive function and noted improved working memory through an interaction with a consolidation mechanism.

### 3.7 ICF-CY domains

The included studies did not explicitly report changes under ICF-CY domains, and the reviewers inferred the appropriate domains through discussion and consensus. Following this process, only two studies reported changes in body structure and function, activities, and participation in ICF-CY domains (36, 38). Most studies reported changes in the body structure and function (31, 33, 35–41, 43); two reported changes in the activities (32, 42), and three studies in the participation domains (32, 33, 35). The reviewers could not comment on the ICF-CY components for a few of the included studies (34, 44) due to the limited availability of information.

## 4 Discussion

This scoping review aims to understand the effectiveness of NIBS in CYP with ABI. Feasibility and exploratory studies suggest that motor function in this population does improve with the use of NIBS. However, it is not possible to determine the actual effectiveness of NIBS in this population due to the variation in study types, small sample size, and the heterogeneity of the patient groups in terms of age of insult, type of ABI, variations in treatment parameters and the outcome measures used. Thus, the findings require cautious interpretation. The authors hypothesised different possible neurophysiological mechanisms for improvements in motor functions, for example, Kirton et al. (31) suggested that the improved motor function was due to TMS influence on the normal ipsilateral hemisphere, creating an imbalance of interhemispheric inhibition and motor cortex inhibition. Nagai et al. (37) noted improved motor control through decreased involuntary movement and proposed that tDCS inhibits the supplementary motor area through the modulation of abnormal excitation of the corticobasal ganglia network, thus potentially influencing the modulation of the corticospinal tract (37). Ryan et al. (35) and Ciechanski et al. (36) reported no specific mechanism apart from neural plasticity for improved motor function using tDCS. The remaining studies (32–34, 38–44) did not suggest any specific mechanism for improvement.

NIBS has been shown to be effective in treating expressive language difficulties, cognition issues and sensory problems, including hearing and vision. However, for similar reasons already discussed, it is impossible to determine the actual effectiveness of NIBS in this area and no specific neurophysiological mechanisms for improvements seen were suggested.

All the studies except one, reported change related to ICF-CY’s body structure and function domain. None of the studies reported how the CYP’s activities and participation changed after TMS or tDCS intervention. For example, Pinchuk et al. study reported a marked reduction in post-lesional migraine headaches, but no specific outcome measures were used to indicate how participation and activities had improved and the associated changes in quality of life (42). It should be noted that the absence of findings related to ICF-CY domains in the included studies is not necessarily linked to the use of NIBS but rather to the lack of its utilisation in guiding rehabilitation outcomes. The reviewers suggest incorporating all ICF-CY domains will help to demonstrate functional improvement in future studies.

This scoping review found that the stimulation time was limited to an average of 20 min in most studies. This was presumably due to convenience and tolerance for CYP being static when receiving NIBS. Pinchuck et al. study used 30–45 min of tDCS stimulation, and they considered the increased exposure time led to a greater stimulation intensity which produced the desired therapeutic response (42). The treatment frequency, intensity, and duration differed for the remaining studies, and in the majority of studies, the rationale for the chosen treatment parameters was unclear. Therefore, deciding the treatment parameters through consensus and different co-production methods with all stakeholders (45), including parents, CYP, neurophysiologists, therapists, and neurologists, should be considered for future studies.

Many studies considered NIBS a useful adjunct, but it was hard to identify the effectiveness due to other confounding factors, including intensive therapy sessions, which may have resulted in neural plasticity. Evidence from children with CP indicates that NIBS promotes rehabilitation effectiveness when combined with intensive interventions such as constraint-induced movement therapy, hand-arm bimanual intensive therapy, treadmill walking, and gait training (46). To induce neural plasticity, repetitive training in conjunction with NIBS is required, and future studies need to consider this to optimise neurorehabilitation outcomes.

Both RCTs included in this review showed that applying NIBS is feasible in the ABI population during the chronic stage (31, 43). Ryan et al. (47) were unsuccessful in recruiting sufficient participants for their feasibility RCT study during the subacute stage of ABI rehabilitation. The authors suggested improved eligibility and retention by approaching children after discharge and providing tDCS on an outpatient basis. The findings of the above studies suggested that acceptability and willingness to try NIBS may be more effective during the later stage of rehabilitation when all other means of rehabilitation have been tried during and after the subacute stage.

Some studies noted minimal side effects (31, 35, 36, 39, 43) and no significant adverse effects were reported. All participants tolerated NIBS intervention well. Our review agrees with other studies that NIBS appears safe for children (48), and this can be considered as an intervention in future studies. Despite having apparently good safety profiles, our review has highlighted that the use of NIBS in the CYP with ABI population is understudied.

There are recognised anatomical and physiological differences between CYP and adults. Skull thickness (46), brain-scalp distances and corticospinal fluid levels are smaller in young children than in adults (49). Therefore, the average current intensity required in the cortex to produce voltage and dosimetry changes is smaller in children than in adults. Due to smaller head sizes, the difference between the anatomical landmarks varies up to 50% in children compared to the adult population (49). Therefore, a smaller TMS coil size is required to stimulate children, and the tDCS montage size needs to be smaller to avoid the shunting effect. The included studies did not indicate whether these differences were considered when stimulating the CYP with ABI using NIBS, and these should be considered in future studies.

It is not currently possible to determine if NIBS has a different therapeutic effect in different types of ABI. For instance, our search found no studies on brain tumour-related ABI, and it may be possible that no studies have been done in this population.

Similarly, it is not currently possible to determine whether NIBS is effective in treating specific symptoms of ABI. For instance, fatigue is a common symptom in CYP with ABI, and it is linked with sleep disturbance, emotional regulation, memory, attention deficiency, and poor academic performance (50, 51). However, there are no studies available which examine NIBS effectiveness in fatigue management.

Given the significant impact of ABI on CYP and their families, more appropriately designed research is required to investigate NIBS effectiveness in the management of various functional difficulties in CYP with ABI.

### 4.1 Deviation from protocol

This scoping review’s initial focus was to assess the effect of TMS primarily on motor function, but the review team subsequently amended this to include tDCS by taking advantage of the already included tDCS-related terms used in the database search, and included other functional difficulties of CYP with ABI, to develop a wider understanding of how NIBS may benefit CYP with ABI. A hand search was planned, but no journals fulfilled the criteria to be considered. Therefore, this was not conducted. Specific details about the NIBS equipment used in the reviewed studies were not included due to the limited availability of information. Comparator/control parameters are not applicable for most of the studies. Therefore, this information was not reported here. The review team had attempted to contact the study authors to verify the collected data through triangulation, which was another deviation from the protocol.

This review has certain limitations. TMS manufacturers were contacted to seek studies using their equipment, but this was not the case for tDCS manufacturers. The review team did not assess the quality of the evidence due to variations in the included study types. The review team could not contact three of the corresponding authors of the conference abstracts to seek further information; therefore, there was no scope to triangulate their findings. It is therefore possible that some of the potential studies that have not been published were excluded. The lack of availability of sufficient data in the studies considered did not allow an in-depth analysis on an individual patient basis.

## 5 Conclusion

The literature describing NIBS interventions in CYP with ABI is scarce. Most available studies have reported positive therapeutic effects, mainly in body structure and functions and activity domains of the ICF-CY. A few studies reported minimal transient adverse effects, but these were tolerated well. An insufficient number of studies, inadequate information reported in them, and small sample sizes limit the ability to conclude how effective NIBS is in improving motor function and other functional parameters in CYP with ABI. Further studies are needed in this area.

## Author contributions

CR: Conceptualization, Data curation, Formal analysis, Methodology, Project administration, Software, Validation, Writing – original draft, Writing – review & editing. VM: Conceptualization, Data curation, Formal analysis, Methodology, Software, Validation, Writing – original draft, Writing – review & editing. DY: Methodology, Resources, Writing – review & editing. PB: Writing – review & editing. JP: Conceptualization, Writing – review & editing. RG: Writing – review & editing.

## Footnotes

^1^ https://osf.io/y5zhx/
